# Supplementary material for: Psychophysiological and psychological responses of touching plant behavior by tactile stimulation according to the foliage type
Source: PLoS One. 2025 Feb 28;20(2):e0316660. doi: 10.1371/journal.pone.0316660 (PMC11870367; doi:10.1371/journal.pone.0316660)
Supplement: S2 File — (DOCX) [file pone.0316660.s002.docx]

**Supporting Information - Data Tables**

**Comparison of SDM during tactile stimulation**

| Tactile  Stimuli | Being relaxed | Being comfortable | Being favorable |
| --- | --- | --- | --- |
|  | Mean±SD | | |
| Soft plants | 5.04±1.55 a | 5.04±1.69 a | 4.97±1.74 a |
| Smooth plants | 5.20±1.09 a | 5.32±1.19 a | 5.31±1.36 a |
| Stiff plants | 3.30±1.36 b | 3.92±1.43 b | 3.91±1.57 c |
| Rough plants | 3.28±1.42 b | 4.33±1.32 b | 4.46±1.45 b |
| F | 54 | 18.3 | 14.18 |
| *p-*value | 0.000*** | 0.000*** | 0.000*** |

*** significant at *p*<0.001, using one-way analysis of variance.

Duncan’s post-hoc analysis (a > b > c) was used for statistical analysis.

Lowercase letters indicate the group to which the activity belonged when performing an analysis using Duncan’s test.

**Variation in SDM responses based on sex, and comparison of responses between sex.**

| Evaluation | Tactile  Stimuli | Male (n=15) | | Female (n=15) | t | *p*-value |
| --- | --- | --- | --- | --- | --- | --- |
|  |  | Mean±SD | | |  |  |
| Being Relaxed | Soft plants | 5.51±1.4 a | | 4.58±1.5 b | 2.965 | 0.004** |
|  | Smooth plants | 5.00±1.0 a | | 5.40±1.1 a | -1.755 | 0.083^NS^ |
|  | Stiff plants | 3.18±1.4 b | | 3.42±1.2 c | -0.851 | 0.397^NS^ |
|  | Rough plants | 3.18±1.3 b | | 3.38±1.4 c | -0.665 | 0.508^NS^ |
|  | F | 38.074 | | 22.680 |  |  |
|  | *p*-value | 0.000*** | | 0.000*** |  |  |
| Being Comfortable | Soft plants | 5.33±1.75 a | | 4.76±1.5 b | 1.632 | 0.106^NS^ |
|  | Smooth plants | 5.09±1.20 ab | | 5.56±1.1 a | -1.874 | 0.064^NS^ |
|  | Stiff plants | 4.51±1.44 bc | | 4.16±1.1 c | 1.278 | 0.204^NS^ |
|  | Rough plants | 4.00±1.52 c | | 3.84±1.3 c | 0.513 | 0.609^NS^ |
|  | F | 7.214 | | 14.359 |  |  |
|  | *p*-value | 0.000*** | | 0.000*** |  |  |
| Being Favorable | Soft plants | 5.47±1.5 a | | 4.47±1.8 b | 2.823 | 0.006** |
|  | Smooth plants | 5.13±1.3 a | | 5.49±1.3 a | -1.241 | 0.218^NS^ |
|  | Stiff plants | 4.84±1.4 a | | 4.07±1.4 b | 2.618 | 0.01* |
|  | Rough plants | 3.80±1.6 b | 4.02±1.4 b | | -0.669 | 0.505^NS^ |
|  | F | 10.238 | 9.153 | |  |  |
|  | *p*-value | 0.000*** | 0.000*** | |  |  |

*** significant at *p*<0.001, using one-way analysis of variance.

Duncan’s post-hoc analysis (a>b>c) was used for statistical analysis. Lowercase letters indicate the group to which the activities belong when performing analysis using Duncan’s test.

^NS^, *, **, *** indicate not significant or significant at *p*<0.05, 0.01, and 0.001, respectively, using independent t-tests.
